# Bayesian Assessment of Newborn Brain Maturity from Two-Channel Sleep Electroencephalograms

**DOI:** 10.1155/2012/629654

**Published:** 2012-03-07

**Authors:** Livija Jakaite, Vitaly Schetinin, Carsten Maple

**Affiliations:** Department of Computer Science and Technology, University of Bedfordshire, Luton LU1 3JU, UK

## Abstract

Newborn brain maturity can be assessed by expert analysis of maturity-related patterns recognizable in polysomnograms. Since 36 weeks most of these patterns become recognizable in EEG exclusively, particularly, in EEG recorded via the two central-temporal channels. The use of such EEG recordings enables experts to minimize the disturbance of sleep, preparation time as well as the movement artifacts. We assume that the brain maturity of newborns aged 36 weeks and older can be automatically assessed from the 2-channel sleep EEG as accurately as by expert analysis of the full polysomnographic information. We use Bayesian inference to test this assumption and assist experts to obtain the full probabilistic information on the EEG assessments. The Bayesian methodology is feasibly implemented with Monte Carlo integration over areas of high posterior probability density, however the existing techniques tend to provide biased assessments in the absence of prior information required to explore a model space in detail within a reasonable time. In this paper we aim to use the posterior information about EEG features to reduce possible bias in the assessments. The performance of the proposed method is tested on a set of EEG recordings.

## 1. Introduction

Abnormal newborn brain development is a life-threatening factor and should be diagnosed as early as possible. Clinical experts can assess brain maturity by analyzing electroencephalograms (EEGs) recorded from sleeping newborns [1–3]. The analysis can take hours of expert work to confidently interpret the EEG. The maturity-related patterns in EEG widely vary during sleep hours as well as between patients and thus finding the regular rules for interpretation of these patterns is a challenging problem [4].

 There are developmental neurophysiology evidences that for healthy newborns the postconceptional age (PCA) normally matches EEG-estimated ages. In cases when the mismatch is observed during two and more weeks, then the newborn's brain maturity is most likely abnormal [2]. Thus, the mismatch between PCA and EEG-estimated age alerts about abnormal brain development.

 In one of the first publications on assessment of brain development [5], the experts have visually analyzed 47 polysomnographic recordings made in 11 PCA groups between 28 and 40 weeks. The polysomnogram included 8-channel EEG, electrooculogram, chin myogram, ECG, and respirogram. In these recordings, the experts have found ten maturity-related EEG patterns. For each EEG recording, the PCA has been estimated based on the distribution of these patterns. The estimates have been found exactly matching the stated PCA in 27.6% of cases. In 59.5% of cases, the matches were within ±1 week, and 85.1% of cases were found matching within ±2 weeks.

 In the aforementioned research, the full polysomnographic information has been used for assessment of developing sleep cycles. As it has been described in [4], these cycles after the 36 weeks are so developed that become visible in EEG exclusively, and so the polysomnograms can be supplementary used for confirmation of the sleep cycles.

 The use of multichannel EEG allows experts to analyze the main patterns related to the brain maturity; however multichannel EEGs recording requires to place multiple electrodes over the scalp. As consequences of that, the preparation for recording becomes more laborious, and sleep more disturbed that causes muscle artifacts more frequently.

 In general, the use of a smaller number of channels enables newborns to sleep more quietly so that the frequency of the artifacts is reduced. The EEG recorded via the central-temporal electrodes, such as C3-T3 and C4-T4, are robust to the muscle artifacts and represent most of the maturity-related patterns (see, e.g., [3]).

 Other authors have attempted to learn brain development models from sleep EEG data recorded from newborns whose maturation was preliminary estimated by experts. In [6], the regression models have been applied to mapping the brain maturity into EEG index. In [7, 8], the classification models have been applied to distinguishing the maturity levels, at least one normal and other abnormal levels of brain development. These approaches aimed at learning a single model providing the maximum likelihood on given EEG data, and thus cannot ensure the maximum accuracy, when the likelihood distribution is affected by noise and its shape is multimodal. Besides, the model selection methodology cannot provide estimates of a full posterior distribution which is required for accurate assessment of the uncertainty in model outcomes.

 In contrast, Bayesian classification enables the uncertainty to be accurately estimated via averaging over areas of high densities of the likelihood (see, e.g., [9–12]). The use of classification models such as Decision Trees (DTs) enables to select features which make the most significant contribution to the classification. Such an ability becomes important when prior information on EEG feature importance is absent. Besides, DT models are understandable for experts. In the case of ensembles, a single DT can be selected for interpretation as shown in [13].

 The results of implementation of Bayesian inference are critically dependent on the prior information as well as on the diversity and areas of model averaging. The use of prior information enables the areas of interest to be specified while providing the necessary diversity in model parameters. When averaging is done over areas of interest with maximum likelihood values, the resultant class posterior distribution is unbiased, and therefore the classification error is minimal.

 Particularly, in many practical cases the prior information on feature importance can be absent so that the areas of interest cannot be explicitly specified and explored in detail (see, e.g., [14, 15]). In [16], it has been shown that selection of EEG features can improve the classification.

 An another approach has been proposed in [17] to cut an ensemble of models while keeping its diversity and performance high. The models with high similarity and high validation errors have been discarded from the ensemble.

 In our previous work [18], we attempted to overcome the aforementioned problem of averaging over areas of interest and proposed a new strategy for Bayesian averaging over DT models. In case of trauma survival prediction, we found that some screening tests make a weak contribution to the model outcome and then assumed that avoidance of such tests will not affect the estimates of the full class posterior distribution. It is important for clinical practice to reduce the number of screening tests required for making reliable decisions. In the experiments we found that the proposed strategy enabled to reduce the number of screening tests, keeping high performance and reliability of predictions.

 In this paper, we aim to further explore the discarding strategy of Bayesian classification on the problem of EEG assessment of newborn brain maturity. We assume that the brain maturity of newborns aged 36 weeks and older can be automatically assessed from the 2-channel sleep EEG as accurately as by expert analysis of the full polysomnographic information as described in [5].

 We expect that using DT models within the Bayesian methodology will enable experts to obtain a set of assessment rules along with an accurate estimate of the full class posterior distribution required to minimize risks. Additionally, the EEG expert will obtain the information about feature importance. We also assumed that the posterior information on feature importance can be used for discarding weak EEG features from the classification models, as we described in [19], and so be used for improving the assessment accuracy.

 The rest of the paper is structured as follows.  Section 2 states the problem of assessment of newborn EEG maturity.  Section 3 describes the methodology of Bayesian averaging over DTs.  Section 4 describes the EEG data used for the experiments, and Section 5 presents the experimental results. Finally Section 6 concludes the paper.

## 2. Problem Statement

Typically, EEG experts assess the newborn brain maturity in terms of PCA measured in weeks. The most of experts agreed that the physical ages of newborns are known in the range ±2 weeks after conception [1, 2, 4, 5]. These ages are often counted from questionnaire of the mother. Ultrasound dating is more reliable than that and normally undertaken on the first and second triple months. The dates are replaced by the ultrasound estimates if the difference in the triples exceeds ±7 days and ±14 days, respectively [20].

 The sleep EEGs are typically recorded via the standard C3-T3 and C4-T4 channels during a few hours. In our case, the EEG recordings have been made by the polysomnograph Alice 3 with a sampling rate 100 Hz. The recordings have been then transformed with the Fast Fourier Transform over each 10 s epochs into the standard spectral power bands: Subdelta (0–1.5 Hz), Delta (1.5–3.5 Hz), Theta (3.5–7.5), Alpha (7.5–13.5), Beta 1 (13.5–19.5 Hz), and Beta 2 (19.5–25 Hz).

 These spectral features were then extended with their absolute and relative values as well as with their variances calculated for each electrodes and their sum. Thus each epoch is represented in a 72-feature space.

 Without the information of feature importance, the Bayesian methodology of model averaging will unlikely ensure unbiased estimates of class posterior distribution. We cannot expect that a multidimensional model space will be explored in detail and the areas of maximum likelihood will be integrated within a reasonable time.

Obviously, information about feature importance could reduce a model parameter space which has to be explored. However in our case, this information is absent and we would have to make an unrealistic assumption that all the EEG features make an equal contribution to the classification. The use of DT models gives us more realistic information on feature importance, and thus the Bayesian averaging over such models will yield the desired information about the EEG features.

We assume that if a feature is rarely used in the DT ensemble, then this feature makes a weak contribution and could be deleted. When there are few such weak features, the portion of DT models using these features is small, and their impact on the outcome is expected negligible.

 In contrast, when the number of weak features is large, the DT models using such features can be disproportionally largely presented in the ensemble. Therefore we could improve the classification results by reducing these DT models. In this paper we aim to explore whether discarding the models using weak EEG features will improve the accuracy of age classification.

 A trivial strategy of using the posterior information for feature selection within Bayesian methodology is to use this information to learn a new ensemble from a data set in which the weak attributes were deleted. This strategy reduces a model parameter space, and therefore this space can be explored in more detail. The other strategy that can be thought of is refining the ensemble by discarding models which use weak attributes. We expect that such refinement can improve the classification accuracy.

## 3. Bayesian Classification

For a DT given with parameters ***θ***, the predictive distribution is written as an integral over the parameters ***θ***:

(1) $$p\left(y\;|\;x,D\right)=\overset{}{\underset{\theta }{\int }}p\left(y\;|\;x,\theta ,D\right)p\left(\theta \;|\;D\right)d\theta ,$$

where *y* is the predicted class (1,…, *C*), *x* = (*x*_1_,…, *x*_*m*_) is the *m*-dimensional vector of input, and **D** are the given training data.

 This integral can be analytically calculated only in simple cases and, in practice part of the integrand, which is the posterior density of ***θ*** conditioned on the data **D**, *p*(***θ*** | **D**), cannot usually be evaluated. However, for ***θ***(1),…, ***θ***(*N*) are the samples drawn from the posterior distribution *p*(***θ*** | **D**), we can write

(2) $$\begin{array}{cc} p\left(y\;|\;x,D\right) & \approx \overset{\text{N}}{\underset{i=1}{\sum }}p\left(y\;|\;x,\theta^{\left(i\right)},D\right)p\left(\theta^{\left(i\right)}\;|\;D\right)\\ & =\frac{1}{N}\overset{N}{\underset{i=1}{\sum }}p\left(y\;|\;x,\theta^{\left(i\right)},D\right). \end{array}$$

The aforementioned integral can be approximated by using Markov Chain Monte Carlo (MCMC) technique as described in [9, 11]. To perform such an approximation, we need to run a Markov Chain until it has converged to a stationary distribution. Then we can collect *N* random samples from the posterior *p*(***θ*** | **D**) to calculate the desired predictive posterior density.

 Using DTs for the classification, we need to find the probability with which an input *x* is assigned by a terminal node to the *j*th class. The DT parameters are defined by *s*_*i*_^pos^, *s*_*i*_^var^, *s*_*i*_^rule^, *i* = 1,…, *k* − 1, where *s*_*i*_^pos^, *s*_*i*_^var^, and *s*_*i*_^rule^ define the position, predictor, and rule of each splitting node, respectively, and *k* is the number of terminal nodes. For these parameters the priors can be specified as follows. First, we can define a maximal number of splitting nodes, *s*_max⁡_ = *n* − 1. Second we draw any of the *m* attributes from a uniform discrete distribution *U*(1,…, *m*) and assign *s*_*i*_^var^ ∈ {1,…, *m*}.

 Finally the candidate value for the splitting variable *x*_*j*_ = *s*_*i*_^var^ can be drawn from a discrete distribution *U*(*x*_*j*_(1),…, *x*_*j*_(*L*)), where *L* is the number of possible splitting rules for variable *x*_*j*_. Such priors allow us to explore DTs which split data in as many ways as possible. However the DTs with different numbers of splitting nodes should be explored in the same proportions [9, 11].

 To sample DTs of a variable dimensionality, the MCMC technique exploits the Reversible Jump extension. To implement the RJ MCMC technique, in [9, 11] it has been suggested exploring the posterior probability by using the following types of moves:

*Birth*. Randomly split the data points falling in one of the terminal nodes by a new splitting node with the variable and rule drawn from the corresponding priors.

*Death*. Randomly pick a splitting node with two terminal nodes and assign it to be one terminal with the united data points.

*Change-Split*. Randomly pick a splitting node and assign it a new splitting variable and rule drawn from the corresponding priors.

*Change-Rule*. Randomly pick a splitting node and assign it a new rule drawn from a given prior.

 The first two moves, birth and death, are reversible and change the dimensionality of ***θ***. The remaining moves provide jumps within the current dimensionality of ***θ***. Note that the change-split move is included to make “large” jumps which potentially increase the chance of sampling from a maximal posterior whilst the change-rule move does “local” jumps.

 The RJ MCMC technique starts drawing samples from a DT consisting of one splitting node whose parameters were randomly assigned within the predefined priors. So we need to run the Markov Chain while a DT grows and its likelihood is unstable. This phase is said *burn-in* and it should be preset sufficiently long in order to stabilize the Markov Chain. When the Markov Chain will be enough stable, we can start sampling. This phase is said *post*-*burn-in*.

## 4. The Proposed Method

We propose a new strategy which aims at discarding the DT models which use weak attributes. First, we apply the Bayesian technique described in Section 2 to the EEG data and collect an ensemble of DT models. Second, we count the posterior probabilities of using EEG features in the ensemble of DT models. These counts give us the posterior information on feature importance.

 Next, we define a threshold value to cut off the EEG features whose probabilities are below this threshold; these features are defined as weak. Then we find the DT models which use these weak attributes. Finally we discard these DT models from the ensemble.

 Obviously, the larger the threshold value, the greater number of attributes is defined as weak, and therefore the larger portion of DT models is discarded. This technique can be evaluated in terms of the accuracy of the refined DT ensemble on the test data. The uncertainty in the ensemble outcomes is evaluated in terms of entropy.

 Having a set of the threshold values obtained in a series of experiments, we could expect that there is an optimal threshold value at which the performance becomes highest. We could also expect to find a threshold at which the uncertainty becomes lowest. In the following section we test the proposed technique.

## 5. Experiments

In our experiments we used EEG data recorded from newborns during sleep hours. The goal of these experiments was to test the proposed method of using the posterior information on EEG feature importance within the Bayesian methodology of averaging over DT models.

 The experiments were run with the set of 72 EEG features representing 686 newborns aged between 40 and 45 weeks so that the number of age groups was six. Each of these groups (classes) included around 100 recordings.

 The Bayesian averaging was run with the following settings. In a burn-in phase, we collected 200,000 DTs, and in a post-burn-in phase 10,000 DTs. During the post-burn-in phase, each 7th model was collected to reduce the correlation between DT models. The minimal number of data samples allowed to be in DT nodes (pruning factor) was set to six. Proposal variance was 1.0, and probabilities of making moves of birth, death, change variable, and change threshold were set to 0.15, 0.15, 0.1, and 0.6, respectively. The performance and uncertainty of the DT ensemble collected in the post-burn-in phase were evaluated within a 3-fold cross-validation and ±2*σ* intervals.

 The rate of acceptance of DT models was around 0.13 in both phases. In the burn-in phase, the size of DTs was stabilized around 30 nodes after 10,000 samples. The average performance (exact match of weeks) was 27.4%. The performance of the DT ensembles varied within 2*σ* interval equal to 8.2%. The entropy of the DT ensembles was 478.3 ± 15.8.

**Figure 1:**
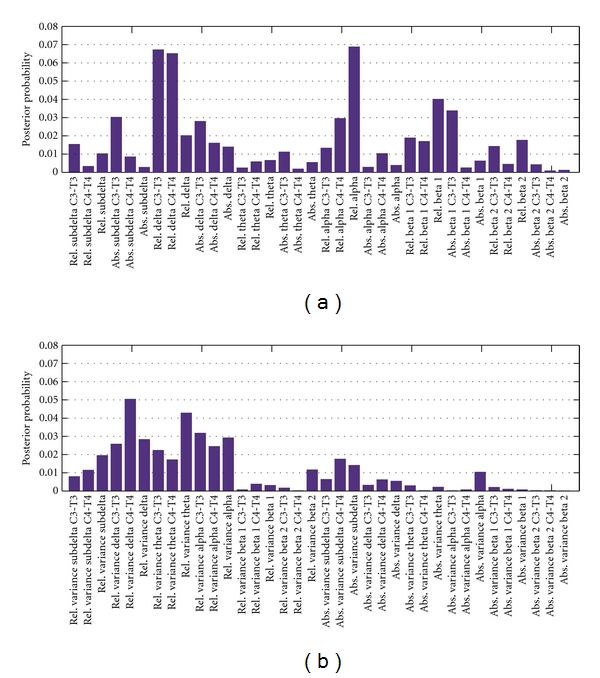
Posterior probabilities of 72 EEG attributes characterising the relative and absolute spectral powers (a) and their variances (b).

 According to the proposed technique, we estimated the importance of all the 72 attributes in terms of the posterior probabilities of using these attributes by the DT models collected in the post-burn-in phase. The posterior probabilities (frequencies) of using the attributes ranged between 0.0 and 0.07 as shown in Figure 1.

 We then gradually increased the threshold value *T* from 0 at steps of 0.001 to 0.005 to define features as weak accordingly to the proposed strategy of feature selection. From Table 1, we can see that at threshold value 0.001 the average number of weak attributes, *k*, was 14, whilst at level 0.005 their number has increased to 31.

 Having found the weak attributes, we applied the proposed technique to refine the DT ensemble. Table 1 shows the number of weak attributes, *k*, versus the threshold values, *T*, within a 3-fold cross-validation. From this table, we can see that the performance *P* of the refined DT ensemble is slightly increased from 27.4 to 29.2 when the threshold is gradually increased from 0.0 to 0.005. At the same time the uncertainty in decisions is decreased from 478.4 to 469.0 in terms of entropy *E* of the ensemble.

 For comparison, we reran the Bayesian classification on the data represented by a set of features excluding the weak ones. From Table 1 we can see that the performance has slightly increased from 27.4 to 29.0 when 23 weak attributes were excluded. The exclusion of 31 attribute has resulted in a decrease in the ensemble entropy from 478.3 to 463.6.

 Overall, both techniques are shown to provide the comparable performances and ensemble entropies. However, the technique of discarding attributes has shown to tend to perform in a larger variation. Within this technique requires rerun the Bayesian classification for each threshold value.

 For comparison, we trained single DTs on the data that preliminary excluded the same weak attributes. The pruning factor was the same as for the Bayesian DTs. The performance of the single DT trained on the original data was 24.6 ± 8.7%. We can see that the discarding of weak attributes leads to a slight increase in the average performance.

**Figure 2:**
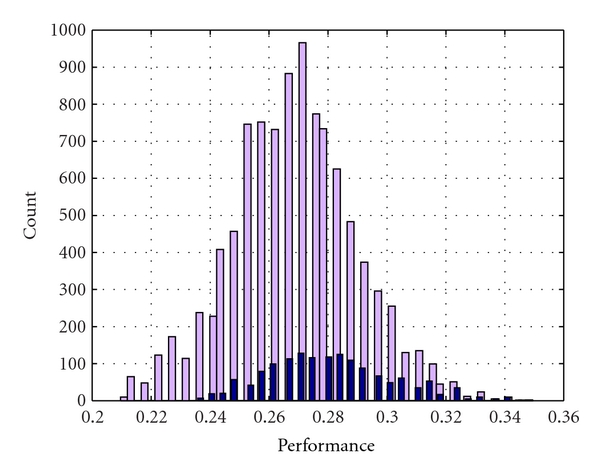
Distributions of performances of DTs included in the original (in gray) and refined (in black) ensembles.

Figure 2 shows the distributions of performances provided by the original and refined DT ensembles. According to the proposed method, the refinement has been obtained by discarding weak attributes with threshold 0.005. We can see that the size of the refined ensemble becomes significantly smaller. Most of the DTs with performance above 32.0% have been kept, whilst most of the DTs with performance below 24.0% have been discarded from the refined ensemble.

**Figure 3:**
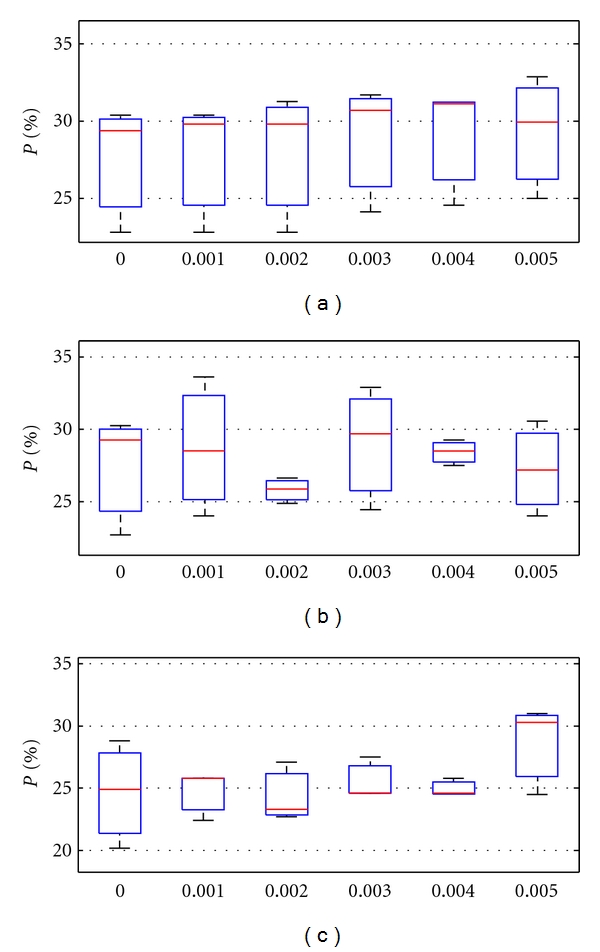
Performances over threshold values obtained with the proposed technique (a), the technique of discarding attributes (b), and single DT (c), respectively.

Figure 3 shows the performances of the techniques over threshold values. Figures 3(a), 3(b), and 3(c) show the performances of the proposed technique of discarding DT models, a technique of discarding attributes, and single DTs, respectively. We can observe that within 2*σ* intervals the average performance of the proposed technique tends to slightly increase when the threshold is growing. As a result of the refinement, the uncertainty intervals estimated in terms of 2*σ* intervals of the entropy *E* are slightly decreased.

Table 2 shows the performance of the expert assessment of EEG maturation described in [5] for 39–43 weeks PCA versus the perforamnce of the Bayesian classification. We can conclude that although the EEG data sets are different, the Bayesian performance, on average, is slightly better than that provided by the experts.

## 6. Discussion and Conclusions

In this paper we explored how the posterior information about EEG features can be employed in order to reduce a negative influence of the lack of exploring the area of interest in detail on the results of Bayesian classification. We assumed that the posterior information about feature importance can be used to find weak EEG features and then proposed a new technique aiming at refining an ensemble by discarding the DT models which use the weak features.

 According to our assumption, in the presence of weak features some DT models included in the resultant ensemble will use these attributes. The larger the number of weak attributes, the greater the negative impact on the classification. We expect that the discarding of models using weak attributes will reduce the negative influence on the classification.

 To test the proposed technique, we used EEG data recorded from sleeping newborns in six PCA groups. We assumed that the brain maturity of newborns aged between 39 and 43 weeks can be automatically assessed from the 2-channel C3-T3 and C4-T4 EEG recordings as accurately as by expert analysis of the full polysomnographic information.

 The use of the additional information will significantly enlarge the dimensionality of a model parameter space and therefore will increase the difficulties of MCMC integration. Thus we cannot expect that additional polysomnographic features or additional EEG channels will significantly increase the assessment accuracy.

 Our experiments have shown that the proposed technique is capable of increasing the performance of Bayesian classification and decreasing the ensemble entropy. We observed that the proposed technique enables DTs with higher performance to be included in the ensemble while discarding the DTs with lower performance. Thus the proportion of DT models included in the ensemble is improved due to decreasing the number of DTs with lower performance.

 We also observed that the MCMC technique makes a candidate model acceptable with different attributes. An accepted model may include by chance a weak attribute even with a small decrease in performance. In the presence of many weak attributes, chances of accepting a model which includes a weak attribute are increased, and this leads to a disproportion of models in the ensemble.

 Typically, a technique of reduction of the data dimensionality by discarding of the weak attributes is expected to improve Bayesian classification by reducing a model parameter space needed to be explored. However this technique requires rerunning the Bayesian classification. The proposed technique was shown to provide the rather comparable performance without the need of rerunning.

 Overall, we conclude that, although the EEG data used in our experiments were from different newborns and the number of recordings was larger, the Bayesian performance, on average, was slightly better than that provided by the experts. Thus our experiments have shown that the brain maturity of newborns aged between 39 and 43 weeks can be automatically assessed from the 2-channel EEG as accurately as by expert analysis of the full polysomnographic information.

## Figures and Tables

**Table 1:** Performance (*P*) and entropy (*E*) of the two techniques versus threshold values (*T*) within 3-fold cross-validation.

*T*	*k*	Proposed technique		Technique of discarding attributes		Single DT
		*P*, %	*E*	*P*, %	*E*	*P*, %
0.001	14	27.5 ± 8.4	478.4 ± 15.8	28.7 ± 9.6	469.0 ± 13.7	24.6 ± 3.9
0.002	18	27.8 ± 9.0	477.7 ± 16.4	25.8 ± 1.7	475.7 ± 16.7	24.3 ± 4.8
0.003	23	28.7 ± 8.2	475.7 ± 15.3	29.0 ± 8.5	474.1 ± 33.9	26.7 ± 3.7
0.004	28	28.9 ± 7.6	471.2 ± 10.3	28.4 ± 1.8	472.4 ± 12.0	24.9 ± 1.5
0.005	31	29.2 ± 7.9	469.0 ± 11.9	27.3 ± 6.5	463.6 ± 26.3	28.6 ± 7.2

**Table 2:** Performances of PCA classification.

Range of PCA	Expert assessment, %	Bayesian classification, %
Exact match	27.3	28.9 ± 7.6
±1 week	54.5	62.6 ± 6.1
±2 weeks	77.3	82.4 ± 4.3
